# Novel non-periodic spoof surface plasmon polaritons with H-shaped cells and its application to high selectivity wideband bandpass filter

**DOI:** 10.1038/s41598-018-20533-8

**Published:** 2018-02-06

**Authors:** Xin Gao, Wenquan Che, Wenjie Feng

**Affiliations:** Department of Communication Engineering, Nanjing University of Science & Technology, 210094 Nanjing, China

## Abstract

In this paper, one kind of novel non-periodic spoof surface plasmon polaritons (SSPPs) with H-shaped cells is proposed. As we all know, the cutoff frequency exists inherently for the conventional comb-shaped SSPPs, which is a kind of periodic groove shape structures and fed by a conventional coplanar waveguide (CPW). In this work, instead of increasing the depth of all the grooves, two H-shaped cells are introduced to effectively reduce the cutoff frequency of the conventional comb-shaped SSPPs (about 12 GHz) for compact design. More importantly, the guide waves can be gradually transformed to SSPP waves with high efficiency, and better impedance matching from 50 Ω to the novel SSPP strip is achieved. Based on the proposed non-periodic SSPPs with H-shaped cells, a wideband bandpass filter (the 3-dB fractional bandwidths 68%) is realized by integrating the spiral-shaped defected ground structure (DGS) etched on CPW. Specifically, the filter shows high passband selectivity (Δ*f*_3 dB_/Δ*f*_20 dB_ = 0.91) and wide upper stopband with −20 dB rejection. A prototype is fabricated for demonstration. Good agreements can be observed between the measured and simulated results, indicating potential applications in the integrated plasmonic devices and circuits at microwave and even THz frequencies.

## Introduction

In nature, surface plasmon polaritons (SPPs) only exists at optical frequencies, in which special surface electromagnetic waves modes can propagate on the interface between metal and dielectric^1,2^. SPPs are highly bound at the metal- dielectric interface and the normal components of electric fields decay exponentially in the transverse direction in near-infrared and visible frequencies. However, in microwave, THz and far-infrared frequency bands, metals behave like perfectly electrical conductors (PECs) without negative permittivity so that they cannot support SPP modes. To overcome the drawback, the concept of spoof SPP metamaterials has been proposed to propagate the plasmonic waves by etching metal surface with periodic subwavelength grooves or holes^3–7^. As we all know, excellent physical characteristics of the so-called SSPPs can be realized by adjusting the geometrical parameters conveniently. Nevertheless, the efficient conversion and transition between the conventional spatial modes and SPP modes is still a challenge. In recent years, several valuable works have been reported to solve the problem in which the matched momentum and impedance can be achieved between common planar transmission lines (such as CPW^7–11^, microstrip line^12–14^, slot line^15–17^) and SSPP waveguide.

The design of microwave devices and circuits is becoming more and more diverse based on different theories, materials, technologies, forms of transmission lines and so on^18–21^. For further study of SSPPs, it is promising to realize plasmonic functional integrated circuits at microwave and even THz frequencies. Lots of researches have been focused on the SSPPs-based devices, including filters^16,22–26^, directional couplers^27,28^, power dividers^29,30^, antennas^31–33^ etc. As we all know, filters are basic and key components in microwave and wireless communication systems, some filters based on SSPPs have also been investigated in recent years. For instance, an ultra-wideband filter with high performance was realized by etching double gratings^16^. However, it was fed by a transducer consisting of step slot lines and gradient corrugated grooves, the port matching for simulation and actual test are thus complex and difficult. Band-pass characteristic was obtained by combining the low-pass feature of the SSPP transmission line with the high-pass feature of substrate integrated waveguide (SIW)^22^. Unfortunately, the additional structures cause more bulky and larger circuit size. In addition, a flexible dielectric film was introduced to produce frequency-selective SSPPs^23^. The structure is composed of two oppositely oriented single-side corrugated strips coupled to two double-side corrugated strips. Another coupled structure constructed by asymmetric grooved strips can also achieve broadband band-pass response^24^, while its out-of-band suppression was further improved. Besides, the frequency-spectrum-controllable SSPPs were proposed by utilizing coupled SRR particles loaded varactor-diodes^25^. The SSPP fields are cut off at the places where the corresponding SRRs are resonant so that multiple controllable rejection bands can be realized. Recently, a compact wideband plasmonic filter with flat-top transmission response has been proposed^26^, however, its upper stopband rejection level needs to be further improved. Most of above proposed filters feature good in-band transmission characteristic but ignore other important performance indexes such as out-of-band rejection and passband selectivity. Therefore, further performance improvement of the filters based on SSPPs is still required.

In this paper, a kind of novel non-periodic SSPPs loaded with two H-shaped cells in the center of the conventional comb-shaped structure is proposed. The cutoff frequency of the new structure decreases a lot and can be adjusted by changing the parameters of H-shaped cells flexibly. More importantly, the proposed structure not only has wide adjustable frequency range but also can realize better impedance matching. Based on the above design ideas, the spiral-shaped DGS etched on CPW is used to feed the proposed non-periodic SSPPs for filter design. A very narrow stopband response can thus be generated at the edge of the passband at the low frequency, while high selectivity of the wideband bandpass filter can be achieved. The proposed structure is compact without adding other components and has good filtering characteristic, indicating potential applications in the plasmonic integrated circuits and communication systems in the future.

## Analysis and Results

### Novel non-periodic SSPPs

**Figure 1:**
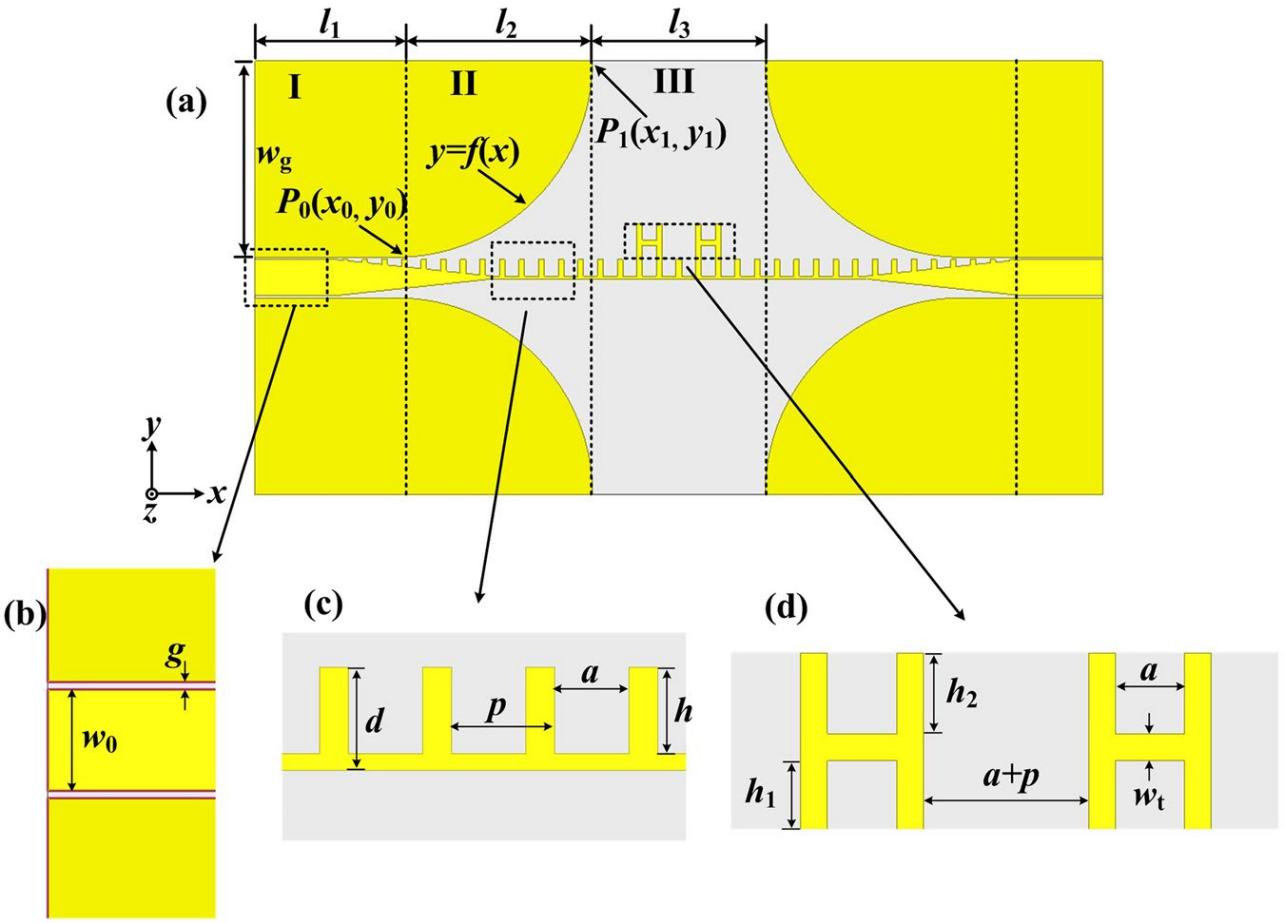
Schematic pictures of the proposed novel non-periodic SSPPs. (**a**) Top view of the proposed structure, in which *l*_1_ = 18 mm, *l*_2_ = 25 mm, *l*_3_ = 22 mm, and *w*_g_ = 25 mm. (**b**) CPW structure, in which *w*_0_ = 4.6 mm, and *g* = 0.3 mm. (**c**) Zoomed view of the traditional comb-shaped structure, in which *d* = 2.5 mm, *p* = 2.5 mm, *a* = 1.8 mm, and *h* = 2.1 mm. (**d**) Zoomed view of two loaded H-shaped cells, in which *h*_1_ = 1.8 mm, *h*_2_ = 2.1 mm, and *w*_t_ = 0.7 mm.

As shown in Fig. 1(a), a new structure consisting of the traditional comb-shaped SSPPs and two loaded H-shaped cells is proposed and fabricated on the Rogers 4003 with relative permittivity of 3.38, electric loss tangent of 0.0027. The yellow part is metal (copper) with thickness of 0.018 mm and the grey part is the substrate with thickness of 1.016 mm. The parameters in Fig. 1(a) are set as: *l*1 = 18 mm, *l*2 = 25 mm, *l*3 = 22 mm, and *w*g = 25 mm. To realize the 50-Ω characteristic impedance at the port, the CPW parameters in Fig. 1(b) are determined as: *w*0 = 4.6 mm, and *g* = 0.3 mm. Here, the conversion part from CPW to the traditional comb-shaped SSPPs is composed of the gradient groove and the optimized curve of flaring ground. To obtain the broadband momentum matching, the depth of gradient grooves varies from 0 to 2.1 mm. The function expression of the optimized curve in part II can be described as:

1 $$y=-\sqrt{{r}^{2}-{(x-{x}_{0})}^{2}}+{y}_{1}\quad \quad ({x}_{0} < x < {x}_{1})$$

where *r* = *y*_1_ − *y*_0_ = *w*_g_. *P*_0_(*x*_0_, *y*_0_), *P*_1_(*x*_1_, *y*_1_) in Fig. 1(a) are established coordinates of the start and the end point of function curve. As shown in Fig. 1(c), for the traditional comb-shaped SSPPs, the total width *d*, the period *p*, the width of metallic grooves *a*, and the depth *h* are chosen as 2.5 mm, 2.5 mm, 1.8 mm, 2.1 mm, respectively. In addition, two loaded H-shaped cells are symmetrically distributed on both sides at the center of the structure. The cutoff frequency of the new non-periodic SSPPs thus decreases a lot with good transmission characteristics in the passband. The parameters of every H-shaped cell in Fig. 1(d) are set as: *h*_1_ = 1.8 mm, *h*_2_ = 2.1 mm, and *w*_t_ = 0.7 mm.

**Figure 2:**
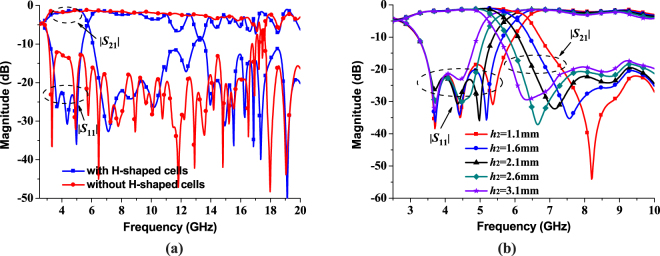
Simulated results of the proposed novel non-periodic SSPPs. (**a**) Simulated reflection coefficients |*S*_11_| and transmission coefficients |*S*_21_| with and without the two H-shaped cells. (**b**) Simulated reflection coefficients |*S*_11_| and transmission coefficients |*S*_21_| of the proposed novel non-periodic SSPPs with different depth *h*_2_ = 1.1 mm, 1.6 mm, 2.1 mm, 2.6 mm, and 3.1 mm.

Figure 2(a) gives the simulated comparison results between the proposed structure with two symmetrical H-shaped cells and the traditional structure without two H-shaped cells. Obviously, it can be seen that the cutoff frequency decreases about 12 GHz (from 17.3 GHz to 5.3 GHz). Remarkably, the return loss is less than −20 dB in the passband for the proposed structure, implying that better impedance matching is achieved. Figure 2(b) shows that the depth of H-shaped cells *h*_2_ can control the cutoff frequency of the proposed novel non-periodic SSPPs and has little influence on the reflection coefficient |*S*_11_|. When *h*_2_ uniformly varies from 1.1 mm to 3.1 mm, the cutoff frequency moves to lower frequency. The 3-dB passband bandwidths are 3.62 GHz, 3.32 GHz, 3.02 GHz, 2.72 GHz, and 2.46 GHz, respectively, with the maximum transmission coefficients as −1.09 dB, −1.12 dB, −1.18 dB, −1.34 dB, and −1.52 dB. To obtain flatter in-band return loss, h2 can be determined as 2.1 mm. Besides the good passband performance, the wide and flexible cutoff frequency range is realized by loading compact H-shaped cells instead of increasing the depth of all the grooves.

**Figure 3:**
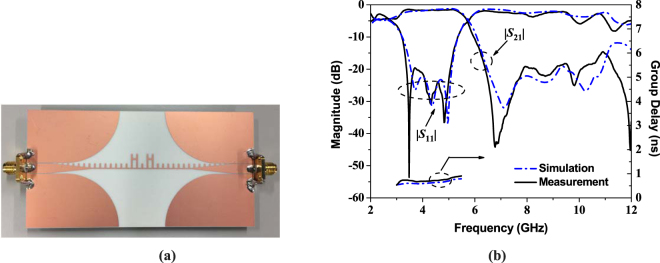
The photograph of the fabricated proposed novel non-periodic SSPPs and the simulation and measurement results of S-parameters and group delay. (**a**) The photograph of the fabricated proposed structure. (**b**) Simulation and measurement results of S-parameters and group delay.

To further verify the accuracy in the microwave frequency, the fabricated novel non-periodic SSPPs with two symmetrical H-shaped cells is shown in Fig. 3(a) for demonstration. The simulated and measured results are illustrated in Fig. 3(b) with a great agreement. The measured 3-dB passband bandwidth is 73.7% from 2.64 GHz to 5.72 GHz with the operating frequency at 4.18 GHz. The proposed novel structure only has around 1.4 dB transmission loss with the return loss |*S*_11_| less than −20 dB in the passband, which indicate a good impedance matching between the CPW and the SSPPs. The group delay is less than 0.9 ns in the whole passband. Furthermore, the fabricated non-periodic SSPPs structure has a wideband out-of-band 18-dB rejection from 6.4 GHz to 10.3 GHz.

### High selectivity wideband bandpass filter

**Figure 4:**
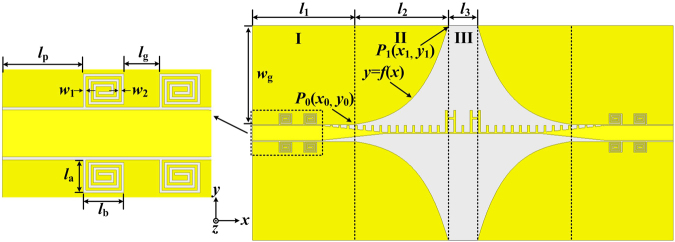
Schematic pictures of the proposed high selectivity wideband bandpass filter. The parameters in the figure are *w*_1_ = 0.25 mm, *w*_2_ = 0.25 mm, *l*_a_ = 3.25 mm, *l*_b_ = 4 mm, *l*_p_ = 8 mm, *l*_g_ = 3. 5 mm, *w*_g_ = 30 mm, *l*_1_ = 31 mm, *l*_2_ = 28. 5 mm, and *l*_3_ = 9 mm, the other unmarked and unexplained parameters of the structure are the same as that in Fig. 1.

In microwave circuits design, DGS is usually applied to change the distributed inductance and capacitance of the transmission line so that the band-stop responses and slow-wave characteristics can be obtained^34^. To improve the passband selectivity of the proposed novel non-periodic SSPPs, the spiral-shaped DGS for CPW is firstly introduced to SSPPs filter design. As shown in Fig. 4, a high selectivity wideband bandpass filter is proposed and fabricated on Rogers 4003 with thickness of 1.016 mm. Compared with the structure in Fig. 1(a), only four symmetrical spiral-shaped DGS are added at the input port and output port of the new structure. According to the enlarged view in Fig. 4, the distance between the first spiral-shaped DGS and the port *l*_p_ is 8 mm and the distance between two spiral-shaped DGS *l*_g_ is 3.5 mm. The parameters of every DGS are set as: *w*_1_ = 0.25 mm, *w*_2_ = 0.25 mm, *l*_a_ = 3.25 mm, and *l*_b_ = 4 mm. In addition, some original parameters are changed: *w*_g_ = 30 mm, *l*_1_ = 31 mm, *l*_2_ = 28. 5 mm, and *l*_3_ = 9 mm, while other parameters of comb-shaped SSPPs and H-shaped cells in Fig. 4 are the same as those in Fig. 1. The function expression of the optimized curve in part II can be described as:

2 $$y=({y}_{1}-{y}_{0})({e}^{kx/({x}_{1}-{x}_{0})}-1)/({e}^{k}-1)\quad \quad ({x}_{0} < x < {x}_{1})$$

where *k* is the exponential factor of the curve and here we choose *k* = 3.2. *P*_0_(*x*_0_, *y*_0_) and *P*_1_(*x*_1_, *y*_1_) in Fig. 4 are established coordinates of the start and the end point of function curve, the following conditions should be satisfied: *x*_1_ − *x*_0_ = *l*_2_, *y*_1_ − *y*_0_ = *w*_g_.

**Figure 5:**
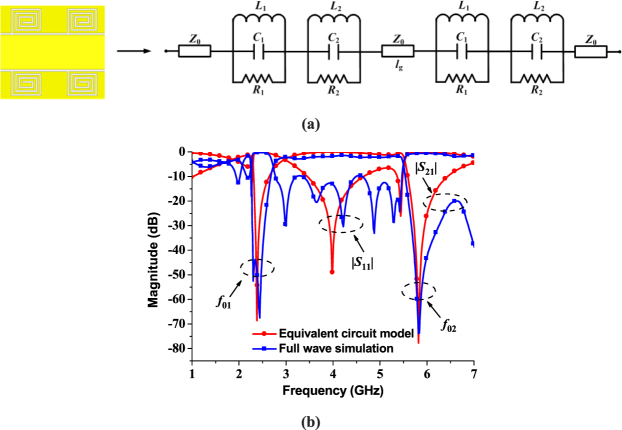
Equivalent circuit of proposed spiral-shaped DGS for CPW, the frequency response of the equivalent circuit model and the full wave simulation result of the proposed wideband bandpass filter. (**a**) The parameters of the equivalent circuit are *Z*_0_ = 50 Ω, *R*_1_ = 6.8 kΩ, *L*_1_ = 1.7427 nH, *C*_1_ = 2.566 pF, *R*_2_ = 7.6 kΩ, *L*_2_ = 0.5126 nH, *C*_2_ = 1.459 pF, and *l*_g_ = 3. 5 mm. (**b**) The comparison of the frequency response of the equivalent model and the full wave simulation result of the proposed wideband bandpass filter.

**Figure 6:**
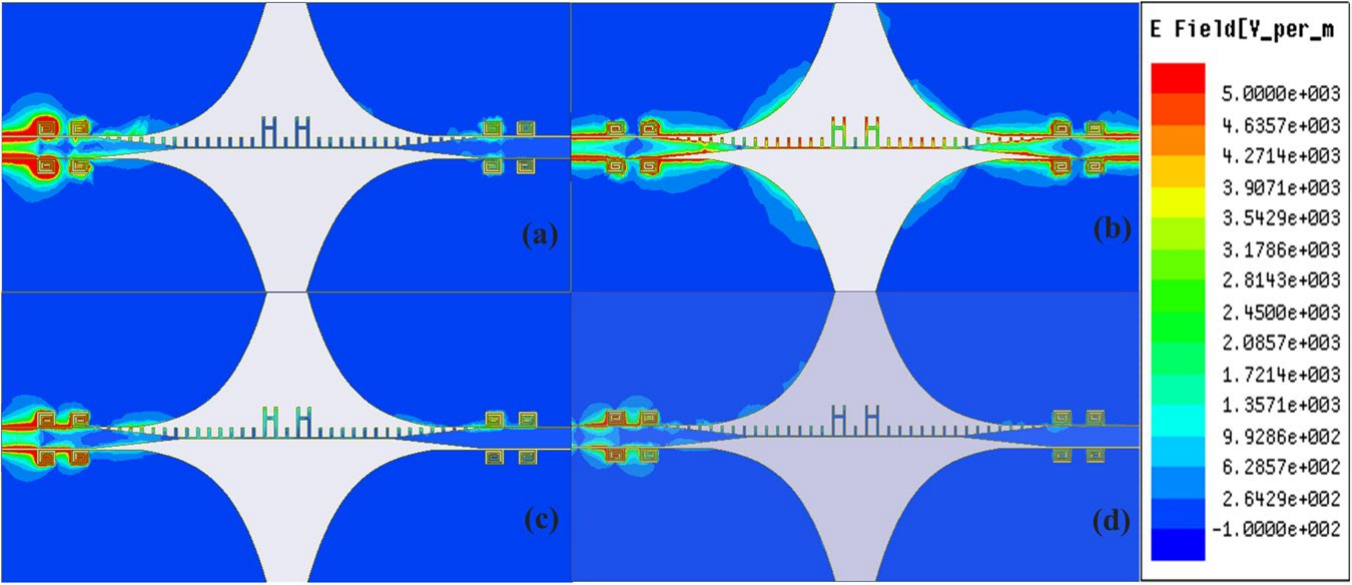
Simulated near-electric-field distributions of the proposed wideband bandpass filter at different frequencies. (**a**) 2.4 GHz (the stopband). (**b**) 4 GHz (the passband). (**c**) 5.8 GHz (the stopband). (**d**) 9 GHz (the stopband).

The band-stop effect of the proposed spiral-shaped DGS resonator can be modelled as a parallel RLC resonance circuit shown in Fig. 5(a). As we all known, changing topologies and geometrical dimensions of the DGS can easily control the equivalent inductance and capacitance of the resonator. Based on transmission line theory and utilization of simulated scattering parameters, the parameters of the resonance circuit for the resonator can be extracted as:

3 $$\begin{array}{c}{C}_{i}=\frac{{w}_{ci}}{2{Z}_{0}({w}_{0i}^{2}-{w}_{ci}^{2})}\\ {L}_{i}=\frac{1}{{w}_{0i}^{2}{C}_{i}}\\ {R}_{i}=\frac{2{Z}_{0}}{\sqrt{1/{|{S}_{11}({w}_{0i})|}^{2}-{(2{Z}_{0}({w}_{0i}{C}_{i}-\frac{1}{{w}_{0i}{L}_{i}}))}^{2}}-1}\quad \quad (i=1,2)\end{array}$$

where *w*_0i_ is the angular resonance frequency, *w*_*c*i_ is the 3 dB angular cutoff frequency and *Z*_0_ is the characteristic impedance of the microstrip line. According to the above formulas, the values of the circuit elements are: *R*_1_ = 6.8 kΩ, *L*_1_ = 1.7427 nH, *C*_1_ = 2.566 pF, *R*_2_ = 7.6 kΩ, *L*_2_ = 0.5126 nH, and *C*_2_ = 1.459 pF. As shown in Fig. 5(b), the two resonance frequency *f*_01_ and *f*_02_ of the equivalent circuit agree well with the two transmission zeros near the passband of the proposed wideband bandpass filter. To show the transmission characteristic intuitively, the simulated near-electric-field distributions of the proposed structure at different frequencies are given in Fig. 6. The frequency points are chosen as 2.4, 4, 5.8 and 9 GHz, which represent the stopband near the first transmission zero, the passband, the stopband near the second transmission zero and the upper stopband, respectively. We can notice that for the stopband shown in Fig. 6(a),(c) and (d), the energy cannot propagate through the center SSPPs structure, while in Fig. 6(b), the energy can propagate intensively and steadily in the passband.

**Figure 7:**
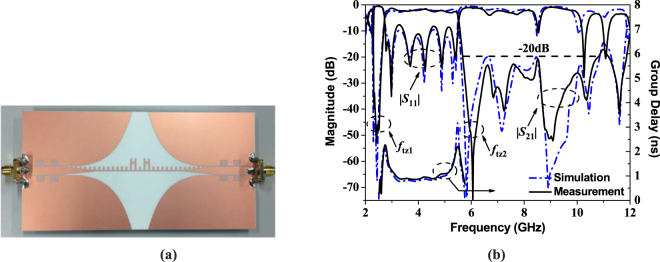
The photograph of the fabricated high selectivity wideband bandpass filter and the simulation and measurement results of S-parameters and group delay. (**a**) The photograph of the fabricated wideband bandpass filter. (**b**) Simulation and measurement results of S-parameters and group delay.

The photograph of the fabricated high selectivity wideband bandpass filter and its simulated and measured results of S-parameters are shown in Fig. 7. Obviously, the measured results agree well with the simulated results in Fig. 7(b). The measured 3-dB passband bandwidth is 68% from 2.72 GHz to 5.52 GHz with the operating frequency at 4.12 GHz. The reflection coefficient is less than −9 dB with the transmission loss around 2.5 dB in the passband. The group delay is less than 0.9 ns in the whole passband. Most importantly, two transmission zeros near the passband at 2.42 and 6.06 GHz lead to high passband selectivity (Δ*f*_3 dB_/Δ*f*_20 dB_ = 0.91). It is widely known that Δ*f*_3 dB_/Δ*f*_20 dB_ is named as square ratio, and it is closer to one, the passband selectivity is better. Meanwhile, the fabricated filter has a wide upper stopband with −20 dB rejection from 5.7 GHz to 10.8 GHz. In addition, the comparison of measured results of our work with previously published reports have been carried out and listed in Table 1. Compared with other wideband bandpass filters based on SSPPs, the passband selectivity of the proposed filter is especially excellent, and more transmission zeros have been realized to improve the upper stopband rejection.

**Table 1:** Comparisons of measured results of our work with the former reports.

Filter structure	Transmission zeros (|*S*_21_|)	Δ*f*_3 dB_ (%)	Δ*f*_3 dB_/Δ*f*_20 dB_	Upper stopband (dB)
[16]	2	161%	0.85	<−20
[22]	4	57.5%	0.84	<−20
[23]	2	43%	0.77	<−10
[24]	3	35.3%	0.73	<−20
[26]-C	3	50.2%	0.8	—
**This work**	**5**	**68%**	**0.91**	**<−20**

Δ*f*_3 dB_, Δ*f*_20 dB_: 3-dB and 20-dB bandwidth of the passband.

## Conclusion

In this paper, a novel non-periodic SSPPs structure is proposed and used for high selectivity wideband bandpass filter design. The non-periodic SSPPs structure is composed of traditional comb-shaped metal strip, two loaded H-shaped cells and CPW transition. Good passband response and impedance matching can be obtained directly, meanwhile its cutoff frequency can be adjusted by the parameters of H-shaped cells flexibly. In addition, the spiral-shaped DGS for CPW is firstly introduced to realize band-stop response on both side of the former passband in the SSPPs structure, and the high selectivity (Δ*f*_3 dB_/Δ*f*_20 dB_ = 0.91) wideband passband filter can be achieved due to the transmission zeros nearby. Both simulation and measurement results have demonstrated the excellent filtering performance for the wideband bandpass filter with the 3-dB bandwidths 68% and wide upper stopband rejection (2.62*f*_0_). The proposed novel non-periodic SSPPs and filter provide application potentials to advanced plasmonic functional devices and integrated circuits in both microwave and terahertz frequencies.
